# Private Sector Investment in Infrastructure in Sub-Saharan Africa Post-COVID-19: The Role of Law

**DOI:** 10.1177/1087724X211059531

**Published:** 2022-04

**Authors:** Augustine Edobor Arimoro

**Affiliations:** Lecturer in Law, Nottingham Law School, 6122 Nottingham Trent University, Nottingham, United Kingdom; and Research Associate, Centre for Comparative Law in Africa, Faculty of Law, University of Cape Town, South Africa

**Keywords:** infrastructure, private sector, public sector, public–private partnership, SDGs, Sub-Saharan Africa

## Abstract

Sub-Saharan Africa (SSA) is the lowest income region of the world with a considerable number of low-income countries. The region is challenged by a massive infrastructure deficit. In recent years, the governments of the countries in the region have expressed the desire to bridge the huge gap in infrastructure assets through a partnership with the private sector using the public-private partnership model. However, the advent of the Coronavirus (COVID-19) pandemic which has resulted in unplanned public sector expenditure poses a new kind of hurdle to climb for states in the region. As such, there is a need for governments in SSA to create and sustain efficient opportunities for private sector investment in infrastructure procurement and maintenance. This article adopted the doctrinal legal research method as well as review of literature in the examination of the role of law in creating a healthy and sustainable business environment for private sector participation in infrastructure financing and operation in a post-COVID-19 era in the SSA region. The article recommends among others, the enactment of legislation to create an enabling environment for raising domestic capital for the purposes of private sector–led public infrastructure procurement as well as the implementation of strategies suited for developing economies to attain successful outcomes in private sector backed infrastructure procurement.

## Introduction

The provision of adequate infrastructure in any given economy is a driver for economic growth (Amadi, 2018). Closing the infrastructure gap is important in order to improve on the quality of life of the population (Lakmeeharan, Manji, & Poeltner, 2020). In this article, infrastructure refers to ‘physical installations such as highways, roads, airports, telecommunication facilities, water supply systems, electricity, waste treatment facilities and the like’ (Kodongo & Ojah, 2016: 106). One of the biggest gaps in infrastructure for Sub-Saharan Africa (SSA) is access to reliable electricity (Holtz & Heitzig, 2021). Given the large scale of infrastructure deficit in SSA across all infrastructure sectors, there is a need for the public sector to create and sustain opportunities for private sector participation in public infrastructure procurement (Chileshe et al., 2020). Also, ‘besides previous issues bedevilling many governments, such as budgetary constraints and political limits to international borrowings for infrastructure…’ (Anago, 2021) the COVID-19 global pandemic creates a difficult situation for the public sector (Anago, 2021). Anago (2021) notes that the impact of the COVID-19 pandemic has made many countries around the globe to ‘pursue aggressive monetary and fiscal policy’ shifts. As a corollary, there will be fewer public funds to invest in public infrastructure while the pandemic lasts and in the short-to-medium term post COVID-19.

SSA fares poorly compared to other regions of the world when infrastructure deficit is put into perspective (Kodongo & Ojah, 2016; (Lakmeeharan, Manji, & Poeltner, 2020). As of 2016, out of the 30 World-Bank-classified-low-income economies across the globe, 25 are in the SSA region (Bond, 2016). In a recent report, it is claimed that SSA is the least industrialised region in the globe and challenged by unreliable power supply problems which compels businesses to spend a huge portion of their revenue in generating their own power via diesel powered plants (Badru, 2020). It is worrisome that the continued underinvestment in public infrastructure assets in SSA might adversely affect the region’s potential (Kodongo & Ojah, 2016). If SSA is to meet its infrastructure goals, the region’s infrastructure deficit must be addressed (Arimah, 2016). Also, given budget constraints and other competing needs for public sector funding such as the cost of running governments and other allocations for recurrent expenditure, the need to employ private sector involvement in infrastructure procurement cannot be over-emphasised.

As noted above, it is not in doubt that the public sector in SSA countries lack the resources bridge the region’s gargantuan infrastructure shortfall. As such, the public authorities in the region need to create as well as sustain an enabling environment to encourage and sustain private sector interests. Partnerships between the public and private sectors can be considered as a tool for growth at the macro level in the economies of SSA (Baker et al., 2019). This article aims to discuss the role law can play in triggering a conducive business environment for public and private sector collaboration in addressing the infrastructure deficit in SSA.

The article is divided into three sections. The first section outlines a new agenda for law and development, the second section examines infrastructure deficit in SSA whilst the third section addresses issues relating to the structuring of legal frameworks and the creation of a sustainable regulatory environment for private sector participation in the procurement of public infrastructure assets. The main aim of this study is to employ law as a tool to create a conducive environment for public–private partnerships in the delivery of infrastructure assets. Indeed, Africa presents an opportunity for investors willing to invest in private sector–led public infrastructure procurement (Kola-Lawal, 2019).

## Law and Development: Does Law Matter in Addressing Sub-Saharan Africa’s Infrastructure Deficit?

The nexus between law and economic development has been the subject of academic discourse for a long time (Ginseng, 2000). Anand (2018) argues that the interplay of law and economic growth will always remain a fascinating subject for legal researchers. If the law is to spur development, the law must be free from uncertainty (Ginting et al., 2018). Lee (2017), in his seminal work on law and development, suggests, and rightly so, that law and development should take into cognisance local variances. Furthermore, there is argument that law and development reform projects that have largely promoted neoliberal ideas have not produced results in developing economies (Lee, 2018). It is therefore important that in discussing law and development in the ‘developing economy context’ that templates used or suggestions for development should be what has worked in other developing economies rather than templates borrowed from developed Western economies.

The essence of law and development theories is to define a connection between law and development via ‘creating a framework within which the role of law in development…’ (Ordor, 2015: 333) can be packaged and suggested to the public authority as a model for advancing development. It is in this light, that this article examines how the law can be used as an instrument to attract private sector investments in public infrastructure procurement in SSA.

In contrast to the ‘one-model-fits-all’ approach of the International Monetary Fund (IMF) programmes in the 1990s, the transition from a state in crisis to a flourishing state can be achieved via different paths (Garcia, 2016). If lessons from other countries like South Korea are anything to go by, then the countries in SSA should consider ways of employing the law to fit their environment to create and sustain conducive business environments for private sector investment in infrastructure.

In the 1970s law and development scholars drew on the analysis of Marx, Durkheim and Weber to develop and activist vision of how the law can be used as an instrument for social change (Ginseng, 2000). The law and development school fizzled out in the 1980s but resurfaced in the 1990s (Ginseng, 2000). The focus of this article is not to discuss the several theories that have been advanced over the years. Rather, this article considers how law can be adopted as an instrument to spur development taking into cognisance the peculiarities of the local environment. An example of how the law has been used as an instrument for development in SSA is the way land is expropriated for infrastructure purposes. Land expropriation is the ‘power of the public authority to acquire legally recognised tenure rights without the willing consent of the tenure holder to serve a public purpose…’ (Tagliarino, 2018). For instance, in Nigeria, the Land Use Act 1978 vests all the land in each state of the federation in the governor of the state.^1^ Thus, upon the payment of compensation to a landowner, the public authority of a state in Nigeria may compulsorily acquire land for purposes of public infrastructure (Arimoro, 2019). As such, the law helps to strengthen the policy of the government to attain infrastructure goals and at the same time ensuring that citizens receive adequate compensation where their land is expropriated (Arimoro, 2019). It follows that law is a tool to achieve policy goals of government and it remains to be stated that in the context of law and development, development represents the transformations of any given economy and social progress experienced (Lee, 2019).

### The Link Between the United Nations’ Sustainable Development Goals and the Need to Bridge SSA’s Infrastructure Deficit

The United Nations (UN) Sustainable Development Goals (SDGs) can only be achieved in the SSA region with a clear policy direction that has its substructure on development-driven legislation (Arimoro & Elgujja, 2019). According to the UN, the SDGs are:

"The blueprint to achieve a better and more sustainable future for all. They address the global challenges we face, including those related to poverty, inequality, climate, environmental degradation, prosperity, peace and justice. The goals interconnect and in order to leave no one behind, it is important that we achieve each goal and target by 2030" (The United Nations, 2019). If the above is anything to go by, SSA has a lot to do to achieve Agenda 2030. In simple terms, the SDGs are targets set out by member states of the UN to be achieved by the year 2030 to ensure global sustainable development (Arimoro & Elgujja, 2019). These goals cover social, economic and environmental development issues including poverty, hunger, health, education, gender equality and clean water. Other SDGs include sanitation, affordable energy, decent work, inequality, urbanisation, global warming, environmental, social justice and peace. Following the setting of these targets, at least 193 countries across the globe have agreed to the targets and have been seeking ways to achieve the goals in the past 6 years (Arimoro & Elgujja, 2019).

Within the context of this paper, the relevant SDGs that can be achieved through bridging the wide infrastructure gap in the SSA include for example, clean water and sanitation, affordable and clean energy, industry, innovation and infrastructure as well as sustainable cities and communities. It is, therefore, vital that SSA countries develop strategies to achieving these targets in partnership with the private sector given that the public sector alone cannot meet the massive demand for infrastructure supply vis-à-vis post-COVID-19 realities. The public–private partnership (PPP) model remains a viable option for the governments in the SSA region.

Arimoro and Elgujja (2019) note that the challenges to realising the goals via PPP include problems of weak institutions, lack of transparency in deals, lack of political will to ensure PPP success and inconsistent government policies among others. This article seeks to address the need to employ the law as an instrument in achieving infrastructure goals that will feed into the attainment of SDGs.

### Law and Development: Lessons from South Korea

Law has a key role to play in economic development. Law is in contemporary times regarded as an integral part of the new conception of development (Prapin & Dhiyathad, 2020). There have been arguments for the Global South to develop its ‘brand’ so to say, of law and development and to show how law can be used to support economic growth (Lee, 2019). However, in order for SSA to develop its strategy for law and development, there is the need to consider what strategies have been tried out in other countries in the Global South that have been successful. It goes without saying, that SSA must not consider templates from the advanced economies of the Global North as such practice in the past have not yielded desired results.

South Korea represents an impressive model for developing economies and especially for countries in SSA on how law can be used as an instrument for development. South Korea has risen from a poor country in the 1960s to become one of the world’s leading economies (Lee, 2019). Around the 1960s, the administration of Park Chung-Hee borrowed a leaf from Japan’s experience and developed a policy that would ensure the diversification of the Korean economy (Kim, 1991). The Koreans developed the private sector by providing several incentives such as tax exemptions and other policies which strengthens private sector growth (Kim, 1991). It needs to be mentioned that at the point Korea succumbed to IMF’s neoliberal changes to policy and law, a consequent reduction in the role of the state in the economy led to the failure of over 3,000 companies and loss of millions of jobs (Lee, 2019). This underscores the fact that in the developing world, the neoliberal approach might not present a suitable template for development.

Another lesson from South Korea is the political will exhibited on the country’s path to development. The problem with many SSA countries is not in the law-making process but in the implementation process of the law. Also, South Korea adopted a degree of flexibility to change. This was exhibited in the degree of the country’s flexible legal frameworks and the political control of the legislative branch of government to enable timely changes in the laws to meet the needs of the country (Lee, 2019).

## An Examination of Sub-Saharan Africa’s Infrastructure Deficit

The inadequate (or lack of) supply of infrastructure (e.g. roads, power supply and potable water) in SSA directly impacts the general quality of the life of the citizens in the region. It is trite that the access to a well-maintained network of roads facilitates the transport of agricultural produce from rural communities to towns and cities. This enables the agrarian rural dwellers to earn income as well as reduce the cost of farm produce and raw materials. In a similar vein, the consistent supply of power helps to reduce the cost of doing business and production. Unfortunately, there is a severe shortage of infrastructure supply in SSA.

On the positive side, it is noted that SSA has made good progress if the growth in the telecommunications sector is anything to go by. In the last 25 years, the sector has been expanding at an appreciable pace across both low-and-middle-income countries (The World Bank, 2017). There has also been growth from 51% to 77% in the access to safe water index (The World Bank, 2017). If the successes experienced in telecommunications can be replicated in other sectors, it would result in improved quality of life for people of SSA. Before the advent of the COVID-19 pandemic, SSA already lagged the rest of the globe in coverage of critical infrastructure classes such as power, road, railway and water infrastructure (Lakmeeharan, Manji, Nyairo, & Poeltner, 2020). The poor state of infrastructure in SSA is due to several factors including unfavourable political and macroeconomic stability, unfavourable conditions for doing business and weak financial markets (Kodongo & Ojah, 2016).

In 2017, the World Bank estimated that for a period between 2016–2040, the global infrastructure deficit will amount to about $15 trillion in 50 countries (Baker et al., 2019). Africa has been assessed to have limited infrastructure whilst the SSA region is noted to be the least developed and lacking in all key infrastructure areas (Goodfellow, 2020). Although it is the responsibility of governments to provide physical assets (such as water, energy and transport facilities) for the populace, most countries can scarcely afford to do so due to competing needs for limited government resources (Wentworth & Makokera, 2015). Most countries in SSA spend between 6 and 12% of their Gross Domestic Products (GDP) annually on infrastructure. Roughly half of the countries spend more than eight per cent of GDP, while only a quarter spend less than five per cent (Wentworth & Makokera, 2015).

### Energy/Power Supply

One of the areas of marked infrastructure deficit in the SSA region is energy. This is a paradox considering the region’s massive hydro energy resource. It is estimated that nearly 600 million people in the region lack adequate access to grid electricity (Lakmeeharan, Manji, Nyairo, & Poeltner, 2020). This accounts for over two-thirds of the world’s population without power. SSA’s power sector is largely underdeveloped. Both residential and industrial sectors experience power shortages resulting in a struggle to sustain GDP growth. The countries in the region having energy access rates exceeding 50% include Cameroun, Ivory Coast, Gabon, Ghana, Senegal and South Africa with the rest of the countries having an average grid access rate of a meagre 20% (Castellano et al., 2015). It is noted that whilst there have been some improvements in the sector to reduce the deficit, the region lags the other regions of the world (Lakmeeharan, Manji, Nyairo, & Poeltner, 2020). For the region to close its energy gap, it is estimated that it would require an expenditure of about $490 billion of capital for energy generation in addition to another $345 billion for transmission and distribution of generated energy (Castellano et al., 2015).

### Transport

The transport sector includes roads, rail, airports and ports facilities. This sector in the SSA region is significantly less developed than in other developing regions of the globe (Bond, 2016). The generally poor state of the sector results in increased transportation costs. In Africa as a whole, road density is 152/km^2^ compared to 211/km^2^ for low-income countries. There is a lack of adequate supply of quality paved roads as the percentage of paved roads in the region is about 13% compared with 25.1% in East Asia and Pacific, and 27% in Latin America and Caribbean regions (Ncube, 2010). Rail networks outside South Africa are generally underdeveloped and poorly maintained. Railways carry only but a fraction of the volumes of two or three decades ago. This is mainly due to poor maintenance as well as ageing networks. Although air transport has grown in the last few decades, there is a need for major upgrades (Bond, 2016). The ports in the region are relatively small compared to peers around the globe. Bond (2016) notes that only the Durban (South Africa) port has an annual capacity equivalent to other developing country ports (4-5 million TEU/year).

### Water

There has been an improvement in access to improved sources of water supply in the SSA region. As of 2015, although more than three-fourths of the region’s population had access to water, this is poor compared to other benchmark regions with rates higher than 90% (Calderón et al., 2018). Also, there is a large disparity in terms of access to water in urban and rural areas. As of 2015, only 67% of rural areas in the SSA had access to water compared to 85% in other developing regions of the world (Calderón et al., 2018).

### Impact of the COVID-19 Pandemic

In the last quarter of 2019, a ‘pneumonia of unknown cause’ which was later named ‘severe acute respiratory syndrome coronavirus 2’ or ‘SARS-SoV-2’ which is now referred to as COVID-19 was initially reported to the World Health Organization (WHO) country office in China (De Groot & Lemanski, 2020). Over the coming months, the virus spread rapidly across the countries of the world leaving serious impacts on economies and travel restrictions. While the origin of the virus can be traced to Wuhan, China, the impact of the pandemic has had a global spread and serious concerns for the SSA region (Ibn-Mohammed, et al., 2020).

The novel COVID-19 pandemic exposed the SSA region’s infrastructure crisis further. Hitherto, there have been concerns about the state of the region’s healthcare infrastructure vis-à-vis population growth (Amewu et al., 2020). As the impact of the pandemic affects every aspect of human life, governments are finding it challenging to cope with ‘unplanned’ COVID-19 expenditure such as the provision of relief items during lockdowns and costs of running ‘makeshift’ COVID-19 healthcare centres among others (Rume & Didar-Ul Islam, 2020).

Whilst the health impact of COVID-19 may not be as serious in Africa compared to the rest of the globe, COVID-19 has nevertheless resulted in major economic disruptions (Ireri & Mathai, 2021). It is envisaged that greater access to infrastructure stocks like energy, transportation (rail and road) especially will help drive down the cost of production of goods and services and create jobs to spur economic growth in the light of several job losses caused by the pandemic (Ireri & Mathai, 2021).

The economies in SSA have been deeply affected by COVID-19 with severe reduction in foreign revenues. The supply and demand sectors of all SSA countries have witnessed massive low levels. For example, there have been fluctuations and downward slope in most cases for commodities including crude oil (Okorie et al., 2020). Oil exporting countries like Nigeria, Sudan, Equatorial Guinea and Cameroun have experienced significant drops in foreign exchange. The same can be said about tourism dependent economies like Mauritius, Seychelles, Kenya and Cape Verde (Okorie et al., 2020).

Looking ahead, there will continue to be limited resources for SSA policymakers aiming to turn around their infrastructure fortunes. The IMF’s SSA regional growth forecast is put at 3.1% for 2021 (International Monetary Fund, 2020). This underscores the need for the public sector to create opportunities for private sector investments in infrastructure. The region is not expected to return to its 2019 levels until around the year 2022. This is so for countries like Angola, Nigeria and South Africa (International Monetary Fund, 2020). According to the IMF (2020), the COVID-19 pandemic has underscored the need for collective action in the face of a worldwide threat.

## Creating an Enabling Environment for Private Sector Investment in Infrastructure

A dearth of adequate financial instruments that support investment in infrastructures as well as governance and regulatory issues are some of the hurdles that bedevil private sector investment in infrastructure in Africa (Arezki & Sy, 2016). In this section of the paper, a partnership between the public and private sectors as well as the legal framework for enabling this model of public infrastructure procurement is discussed.

### Public–Private Partnership

In the modern era, ‘it is unfeasible for the government of any country to supply public infrastructure to match the demand by its citizens’ (Arimoro, 2020: 1). Budget deficits and competing demands for public sector expenditure necessitate a partnership between the public and private sectors to manage the supply and demand for public infrastructure assets. It, therefore, behoves on the public sector to develop and implement policies and strategies that would facilitate public infrastructure procurement and funding to improve the lives of the citizenry (Arimoro, 2020). Consequent upon the foregoing, a collaboration with the private sector to fund infrastructures could be relied on as the alternative to tradition public procurement. The main objective of public–private partnership (PPP) around the globe is basically to ‘ensure the delivery of well-maintained cost-effective public infrastructure…by leveraging private sector expertise and transferring risk to the private sector’ (National Treasury PPP Unit, 2007: 4).

There has not been, and it is difficult to imagine that there would one day be a universal definition of the term ‘PPP’ (Arimoro, 2020). There is, however, a consensus that PPP is a collaboration between the public and private sectors for the delivery of public infrastructure (Nwangwu, 2016). But this does not clearly explain what a PPP is. It is important that in defining what a PPP is, that it is not confused with other arrangements between the private sector and the government. For example, a PPP is not the same thing as privatisation. In a privatisation arrangement, the government divests all or some of its shareholdings in a public enterprise to a private sector entity. Privatisation is the transfer of ownership rights in a state-owned enterprise to the private sector (Davidson, 2014). Again, an arrangement that merely involves the private sector in the management of the public facility is not a PPP but a management contract as this does not involve any form of construction whether as a new (greenfield) or a rehabilitation (brownfield) project. In the same vein, a service arrangement that requires the private sector to render service such as a cleaning service in a public facility is not a PPP but a service contract (National Treasury PPP Unit, 2007).

In their seminal work, Pierson and McBride (1996) have provided a guide for distinguishing PPP from other similar arrangements between the public and private sectors. They state that to distinguish PPP, the following elements must be present: first, there has to be a transfer of land, property or facility by the public authority to the private sector entity; second, the private sector entity builds or renovates the facility; fourth, services are provided by the private entity for a defined period and fifth, the private sector entity agrees to transfer the facility to the public authority at the termination of the arrangement. It, therefore, follows that where any of these elements is missing, the arrangement is not a PPP (Arimoro, 2018) in the context of the discussion in this article. Perhaps the view expressed by Pierson and McBride influenced the definition that has been offered by Yescombe. According to Yescombe (2017: 3), a PPP is:

- A long-term contract between a public-sector entity (the ‘public authority’) and a private-sector entity (‘the project company’) involving significant risk transfer to the private sector.
- Under the contract, the project company is responsible for the design, building (or upgrading), finance, operation and maintenance of public infrastructure (‘the asset’).
- The private sector financial investment is repaid from revenues generated by the asset under the terms of the PPP contract, payable either by users of the asset (e.g., toll-road concession) or by the public authority (e.g. government accommodation) or a combination of the two.
- At the end of the PPP contract, the asset usually remains in, or reverts to, public-sector ownership. (Thus, a PPP is not the same thing as privatisation).

A PPP is, therefore, a long-term binding contract between the public authority and a private sector consortium for the design, financing, construction and management of public assets for a fixed term of usually between 20 and 30 years within which period risk is transferred to the private sector and at the termination of which the asset or assets is/are transferred back to the public authority (Arimoro, 2020). Typically, the main features of PPPs are that they are cooperative and contractual relationships with shared responsibilities.

### Legal Framework for Public–Private Partnership

PPPs are by their nature, very complex arrangements and are usually regulated by law (Joković, 2018). Legal measures must be put in place to efficiently implement PPP policies especially in developing regions of the world (Nwangwu, 2012). Unlike a traditional procurement, where the public authority contracts a private company as a supplier of assets or services, in a PPP, the relationship moves beyond that of a client-supplier to a partnership between the public authority and the private consortium. The mandate of the private consortium is to deliver an infrastructure project over the long term in return for a reward in form of charges or tolls (Arimoro, 2020).

The PPP framework refers to ‘the policy procedures, institutions, and rules that together define how PPPs will be implemented – that is, how they will be identified, assessed, selected, budgeted for, procured, monitored and accounted for’ (The World Bank, 2014: 65). As such, the existence of an appropriate legal framework is a requirement to creating an environment that fosters private investment in infrastructure.^2^ Most countries with successful PPP experience often do so anchored on a viable PPP framework (The World Bank, 2014). It is important that PPP laws remain dynamic and responsive to the needs of the local economy. Against this backdrop, there is no point borrowing templates from advanced economies as those frameworks are usually tailor-made for developed economies and often do not work where institutions are not as developed and independent as they are in the Global North.

The establishment of a clear PPP framework for PPPs in any given economy is an indicator that there is a commitment on the part of the public authority to ensure that PPPs succeed (The World Bank, 2014). The framework should outline how projects are to be initiated, procured, implemented, managed and operated. Without a doubt, clarity will help get and sustain investor interests in PPPs in the SSA region. According to Delmon (2014) a conducive PPP framework must aim to achieve the following:

- the political will to pursue PPP, and the legal and regulatory regime appropriate to enable and encourage PPP.
- selection, design and development of ‘good’ projects – the most appropriate and feasible projects for PPP.
- allocating risk to the private sector while insulating investors from those risks best borne by the contracting agency or the Government.
- ensuring that the financial markets are in a position (legally, financially and practically) to provide the project with the investment it needs (debt, equity and otherwise), including by providing Government support.

Furthermore, it is a lot better to adopt a simple framework for PPP to make the process clear for all stakeholders. The essence of having a framework, among others, is so that the government can establish what the PPP policy is, pass the necessary laws and regulation, establish a PPP unit to administer the process, select and develop a pipeline of projects to be executed using the PPP model and monitor the process and projects. The legal framework refers to the laws and regulations that underpin the PPP programme in any jurisdiction (The World Bank, 2014).

There are key areas that the public authority must seek to address vide a legal framework. These areas must be addressed to provide local and foreign investors assurance that the government intends to be bound by the PPP contracts as this constitutes a major source of worry in developing economies (Delmon, 2014).

The legal framework must specify and describe which government agencies have the power to perform which functions associated with a PPP project. Where a party performs an act outside of that party’s legal rights, it would be judged *ultra vires* and would be void under the law. A very good example is that of sub-national governments in Nigeria. None of the 36 federating units in Nigeria has the power to enter contracts dealing with inland waterways, federal roads or aviation among other items listed in the Exclusive Legislative List (Schedule II) of the 1999 Constitution of the Federal Republic of Nigeria (as amended). Even though some state governments like Akwa Ibom and Delta have constructed airports within their jurisdictions, a prospective investor should be concerned about the legality of such a contact if procured as a PPP.

The procurement process must be clear, straightforward and simple. The importance of public consultation in the build-up to the signing of a PPP contract cannot be overemphasised. In SSA, there has been a trail of criticisms about the alienation of key stakeholders in the PPP procurement process. For example, in South Africa, the process has been said to be ‘riddled with corrupt practises in what has become known as *tenderpreneurship’* (Fombad, 2013: 11). The selection process for PPPs must be seen to be fair by all parties involved. There should also be a process available to challenge the award of projects to winning consortiums (Delmon, 2014).

The legal framework for PPPs should take into cognisance land rights and acquisitions as this could potentially be of great concern for PPP promoters and investors (Arimoro, 2020). Land expropriation is understood to be one of the bottlenecks militating against the execution of PPP projects in some developing economies.

PPP investors are concerned about return on investments and as such, end-users of PPP facilities may be expected to make payments in form of charges or tolls for the use of PPP assets/facilities. The legal framework should define how tools and charges are to be collected and adjusted over time. Also, it must be clear how the project company can enforce its rights to collect tariffs through penalties or disconnections (Delmon, 2014).

Since PPPs are complex contracts, disputes are inevitable. Failure to address such disputes early and cheaply may negatively impact the PPP regime in any given economy. Generally, parties to a PPP contract/project would prefer to submit any disputes that may arise during the tenure of the contract to arbitration. This is because it is cheap, flexible and offers ease of award execution.

Also, given the fact that lenders recourse in the event of a PPP failure lies in the project company, it is not uncommon for lenders to want to exercise a right of control of the project company’s assets to protect the lender’s assets. It is, therefore, significant to include regulations to deal with the taking over of security over different projects.

### Measures for Unlocking Domestic Funding

Given the impact of COVID-19, governments in the SSA region will struggle to cope with meeting the demands for more infrastructure assets (Anago, 2021). Also issues regarding foreign exchange risks (Moosa, 2004) will mean that foreign investors might not be attracted to investing in the SSA region. This is so because charges or tolls are usually denominated in the local currency even though the funds invested in infrastructure projects are usually denominated in United States Dollars. What this means is that currency fluctuations especially when they go south (i.e. when the currency of a country is devalued) is not in the interest of foreign lenders/investors. There is, therefore, the need in the post-COVID-19 era to create opportunities for domestic funding of PPP projects.

As against borrowing funding templates from advanced economies in the Global North, the governments in the SSA region should develop strategies that would work in their region taking into cognisance local peculiarities. Attracting more private investment for infrastructure in SSA is a huge take especially as only the respective governments of Angola, Ghana, Nigeria and South Africa have made significant efforts to deal with their infrastructure deficits (Möykkynen & Pantelias, 2020).

A first step would be enacting primary or secondary legislation to allow fund managers to set up infrastructure funds. Infrastructure funds managed by the fund managers in the various SSA economies can become an avenue for channelling pension fund investments into infrastructure development (Newell et al., 2011). Further, the infrastructure funds, unlike the current practice where only high-net-worth investors can subscribe to such funds, there should be low entry thresholds for subscribers. This is to enable low-to-middle-income earners to participate in infrastructure finance. High entry infrastructure funds require investors to contribute large sums of money which usually make them the preserve of high-net-worth investors. The argument of this article is that low entry mutual funds with a mandate for investments in infrastructure should be set up in the various SSA economies. Infrastructure funds can serve as an alternative investment instrument for investors and pension fund managers that wish to diversify their portfolios or who desire to have low risk instruments as against the volatility of equity assets/securities.

One of the challenges in the SSA region in financing long-term infrastructure projects is the inability of domestic banks to provide long-term loans. This is not unconnected to poor savings culture in the SSA region. Given the emphasis in this article for developing economies to take a cue from other developing economies instead of the advanced economies of the Global North, the SSA region may do well to borrow a leaf from the Indian model of infrastructure financing. India has a reasonable high savings rate. It is estimated that the saving as a portion of GDP is 22.3% for household, 7.2% for corporate and 1.3% for the public sector. To be able to channel these savings into financing infrastructure, the Government of India passed several legislation to facilitate among others, the Infrastructure Debt Fund, Amendment of the IRDA Investment Regulations 2013, and enactment of a new Land Acquisition Act (Agrawal, 2020).

As noted by Anago (2021), the SSA region can look towards channelling funds managed by pension fund managers to developing infrastructure assets. Infrastructure funds are one of the asset classes that could be appealing to pension fund managers in search of yield and the fact that infrastructure funds provide better protection against market volatility (Inderst, 2009). To facilitate investment of pension fund assets in infrastructure funds, the governments in the SSA must put in place ‘the right governance structure, effective regulation and availability of sufficient risk-mitigating instruments…’ (Egbuna et al., 2018: 20). Governments should take steps to remove constraints that prevent investing pension assets in infrastructure assets and put in place effective risk management measures. This can be achieved by implementing a predictable policy framework (Sy, 2017) that is based on a legal foundation.

In the SSA region, South Africa has the most advanced financial market and can serve as a template for the other SSA economies. For example, South African pension fund Eskom Pension and Provident Fund invested about $30 million in infrastructure projects via Dubai-based Abraaj Group over 10 years ago. In 2011 and 2014, the fund invested $5 million in a company that builds mobile phone towers through London’s Helios Investment Partners LLP (McGroarty, 2015). This is made possible because of South African legislation that allows for pension funds to invest in infrastructure assets.

Another asset class to be considered for domestic infrastructure funding is the opportunity for setting up infrastructure bonds. Such bonds might be reflected in pension fund portfolios and other private wealth portfolios managed by investment management companies (Inderst, 2010).

### Law as a Catalyst for Reform

Development economists and policymakers consider law as a vital ingredient in implementing state policies. Therefore, reforming the legal system itself and the strengthening of laws to ensure contract rights, transparency and good governance have become a focus for policymakers around the world in contemporary times (Kennedy, 2013). Economic theories are, therefore, transmuted into objectives to be pursued by policymakers and translated into legal instruments. While the role of law as an instrument of economic growth has been established, the conscious use of the instrument of law to bring about economic development in the SSA region is long overdue (Baderin, 2010). Given the above assertion, it is imperative to initiate development-driven laws to achieve growth and economic development.

Consequent upon the foregoing, the countries in the SSA region should identify areas where they require making legal reforms to allow for needed changes to facilitate private sector-led infrastructure development.

## Conclusion

This article analysed the importance of infrastructure in economic growth and the role that infrastructure plays in enhancing the quality of life of citizens in every country in the world with a focus on the SSA region. It has been noted that the SSA region lags the rest of the other regions of the world as far as key infrastructure classes like energy, transport and water supply is concerned.

Before the advent of the COVID-19 pandemic, the SSA region already suffers from a gargantuan infrastructure deficit especially in the critical sectors. With the COVID-19 situation, there is an increased burden on the public authorities in SSA to match infrastructure demand with supply. This additional budgetary requirement in the face of fiscal constraints makes an already bad situation worse. The outlook for foreign investment in infrastructure in the SSA region is bleak. This is in addition to the challenge of foreign exchange risks given that investments in infrastrastructures are denominated in US dollars whilst return on investment is usually denominated in the host country’scurrency. These make foreign investments in the SSA region in a post-COVID-19 era unattractive in the short-to-medium term.

It is therefore, incumbent on the public authorities in the SSA region to create opportunities for unlocking domestic finance for private sector investments in infrastructure financing. Given the massive population size in the region, it is argued in this paper that it is not all doom and gloom as infrastructure deficit is a blessing in disguise as it presents investment opportunities for investors. For example, if the success recorded in telephony in the SSA region is taken into reckoning, investments in other sectors can be rewarding to investors and will help provide the needed infrastructure to improve on the quality of life for the populace in the region.

The public authorities should develop policies that will allow for fund managers to create infrastructure mutual funds with low entry thresholds to allow low-to-middle income earners to subscribe to the funds. The current available infrastructure funds that allow only high-net-worth individuals to invest do not take into reckoning the effect that crowd funding can have with several persons having the opportunity to invest in infrastructure rather than only a few. SSA is one of the most populated regions in the world and if these numbers are efficiently used investment-wise, significant funds can be raised to fund public infrastructure projects in the post-COVID-19 era.

There is the need to encourage savings. If the examples from Japan, South Korea and India are anything to go by, then the public authorities in SSA should develop measures to ensure that apercentage of income earned is saved by households, the corporate world and by the various tiers of governments. This can be achieved by the enactment of relevant legislation.

## Notes

1 Land Use Act 1978 section 1.

2 UNCITRAL Legislative Guide on Privately Financed Infrastructure Projects UN 2001 A/CN.9/SER.B/B4 23.
